# Genetic characterization of maize germplasm derived from Suwan population and temperate resources

**DOI:** 10.1186/s41065-018-0077-1

**Published:** 2019-01-10

**Authors:** Xun Wu, Angui Wang, Xiangyang Guo, Pengfei Liu, Yunfang Zhu, Xiushi Li, Zehui Chen

**Affiliations:** Institute of Upland Food Crops, Guizhou Academy of Agricultural Sciences, Guiyang, 550000 Guizhou China

**Keywords:** Maize germplasm, Genetic characterization, Suwan population

## Abstract

**Background:**

The Suwan population is a well-known maize germplasm that has greatly contributed to the development of maize breeding in tropical and subtropical regions, especially in southern China. Inbred lines derived from the Suwan population always contain stronger resistance and extensive adaptability in different environments. To evaluate the genetic character of inbred lines derived from the Suwan population, a panel including 226 inbred line derived from the Suwan population and temperate resources was assembled and genotyped by using MaizeSNP50 BeadChip, which contained 56,110 genome-wide single nucleotide polymorphism (SNP) markers. This panel contained 98 temperate inbred line and 128 lines derived from the Suwan population.

**Results:**

The results showed that high genetic diversity was found, with PIC and GD to be 0.67 and 0.60, respectively. In addition, two novel subgroups were identified, with representative inbred lines as HCL645 and Ki32, respectively. One acknowledged heterotic group of Iowa Stiff Stalk Synthetic (SS) was also identified in this study. This study can provide some additional scientific evidence for heterotic group division and use in maize. Additionally, lower linkage disequilibrium (LD) levels and weaker genetic relationships were found, with an average LD level of 41.15 kb that varied from 3.5 to 96 kb. A total of 82.8% of paired relative kinships ranged from 0.05 to 0.28.

**Conclusions:**

These results would not only facilitate maize breeding practices in tropical and subtropical regions, but also revealed that this panel can be used in dissecting the genetic basis of complex quantitative traits’ variations by using genome-wide association studies (GWAS).

**Electronic supplementary material:**

The online version of this article (10.1186/s41065-018-0077-1) contains supplementary material, which is available to authorized users.

## Introduction

Maize (*Zea mays L.*) rapidly spread throughout the world after its domestication from teosinte in tropical Mexico [14]. Many local landraces or farmer’s varieties were formed owing to the repeated introduction, artificial selection, and adaptation to different environments. Some representative inbred lines with different origins were produced, and relevant hybrids were released. To continuously improve the performance of hybrids adapting to partial ecological environments effectively, heterotic groups and heterotic patterns were clarified [11, 12, 26], which played important roles in maize commercial breeding programs. However, to date, less than 5% of germplasm is used in maize breeding around the world, and less than 1% is used in the U.S. [4].

A few classical inbred lines were repeated used to develop new lines and excellent hybrids in temperature conditions, which contributed mostly to maize hybrid breeding [20]. i.e., B73, PH207, Mo17 [13], and Huangzaosi (HZS) [26].For instance, most lines belonged to Stiff Stalk (BSSS) and were derived from B73, and approximately 35% of Pioneer’s hybrids were from BSSS. Mo17 was common used in all U.S. seed companies, and more than 45% of Pioneer’s commercial hybrids contain the pedigree of Mo17 [19]. In the Huang-Huai River region of China, HZS is widely used during the maize breeding process, has derived more than 40 elite lines and 80 hybrids and has greatly contributed to the industrial development of Chinese maize. For example, the maize hybrid ‘Zhendan 958’ (Zheng58 × Chang7–2) has been grown in an area of more than 35 million ha in China [25].

In addition, Tuxpeño is one of the well-known maize germplasms that has greatly contributed to the development of tropical maize breeding programs [1]. Tuxpeño maize pools often contain a broad genetic base and show larger diversity than that in temperate populations. Tuxpeño germplasm’s productivity and crossing it with the race ETO result in Tuxpeño-ETO heterotic patterns in tropical maize breeding, which are widely used in Thailand, Colombia, and Mexico [23]. However, during the Tuxpeño maize pools’ ultilization process, its lower resistance to some diseases and insect pests in tropical regions was found, which limited its widely use. Therefore, some improvements were done during maize breeding practice. Since the 1960s, 36 accessions have been collected across the world, and relevant agronomic traits have been evaluated in order to improve maize adaptation under Thailand’s cultivation conditions. Based on the results of data analysis, one synthetic variety of Suwan1 with excellent performance was released through modified ear-to-row selection methods, which showed better performance in tropical regions [21], and rapidly spread to tropical and subtropical regions as a result. The heterotic pattern by crossing lines from Suwan1to temperate resources became famous and was widely used in subtropical regions, especially in southwest China. However, knowledge on the genetic differences between Suwan population and temperate maize germplasm is still limited, which remains one of the impediments to its increased use in breeding programs.

For decades, many marker-based studies have addressed genetic diversity, population structures, and genetic relationships by using different panels and markers Wang et al. [24] showed that only four heterotic groups were identified when integrating only the Chinese elite lines from temperature regions together, which was smaller than that found by using tropical and temperate lines together [11, 28]. In Yan’s studies, 632 lines from temperate and tropical/subtropical public breeding practices were used: it contained 351 tropical/subtropical lines from the Maize and Wheat Improvement Center (CIMMYT) and showed much higher genetic diversity than that in the temperate panel [11]. However, fewer markers and resources of maize germplasm were used in other reports, which would provide limited information when dissecting the potential genetic diversity in maize germplasm.

By using the genotyping by sequencing (GBS) method, Romay et al. [17] evaluated the genetic characters of 2815 accessions in the USA national maize inbred seed bank. Based on 681,257 SNP markers, many more rare alleles were identified, but only a modest amount of the available diversity was found in the commercial germplasm. Strong population stratification was identified, with a small number of key lines as the center of one cluster. This genotypic information provided abundant insight into maize genetic diversity around the world. However, most lines used in previous studies were publicly available, representing historical recombinants during the maize domestication and improvement progress. Along with the development of private commercial maize breeding, more and more new elite inbred lines were produced and widely used in modern maize breeding practice. This was especially true for the Suwan population, which came from tropical regions, and derived many more elite lines widely used in tropical/subtropical maize breeding program, such as Ki32, QR273, and QB446. Therefore, collecting one naturally diverse panel that contained the latest lines derived from temperate and Suwan resources that are widely used in modern breeding programs is better for in-depth study of the genetic character of maize germplasm. Based on this panel, we dissect the genetic relationship between pair-lines by using the high-throughput genotyping method, which is important not only for expanding gene pools to accelerate maize breeding progress but also for providing additional information for evaluating whether this panel is suitable for genome-wide study (GWAS) in analyzing variation of complex traits.

In this study, 226 accessions derived from the Suwan population and temperate resources were selected and genotyped by using the MaizeSNP50 BeadChip, including approximately 56,110 single nucleotide polymorphism (SNP) markers. The objectives were to evaluate the genetic diversity, population stratification, relative kinship, linkage disequilibrium (LD) level, and whether this panel is suitable for GWAS of agronomic traits’ variation.

## Results

### Model-based population structure and relative kinship

**Fig. 1:**
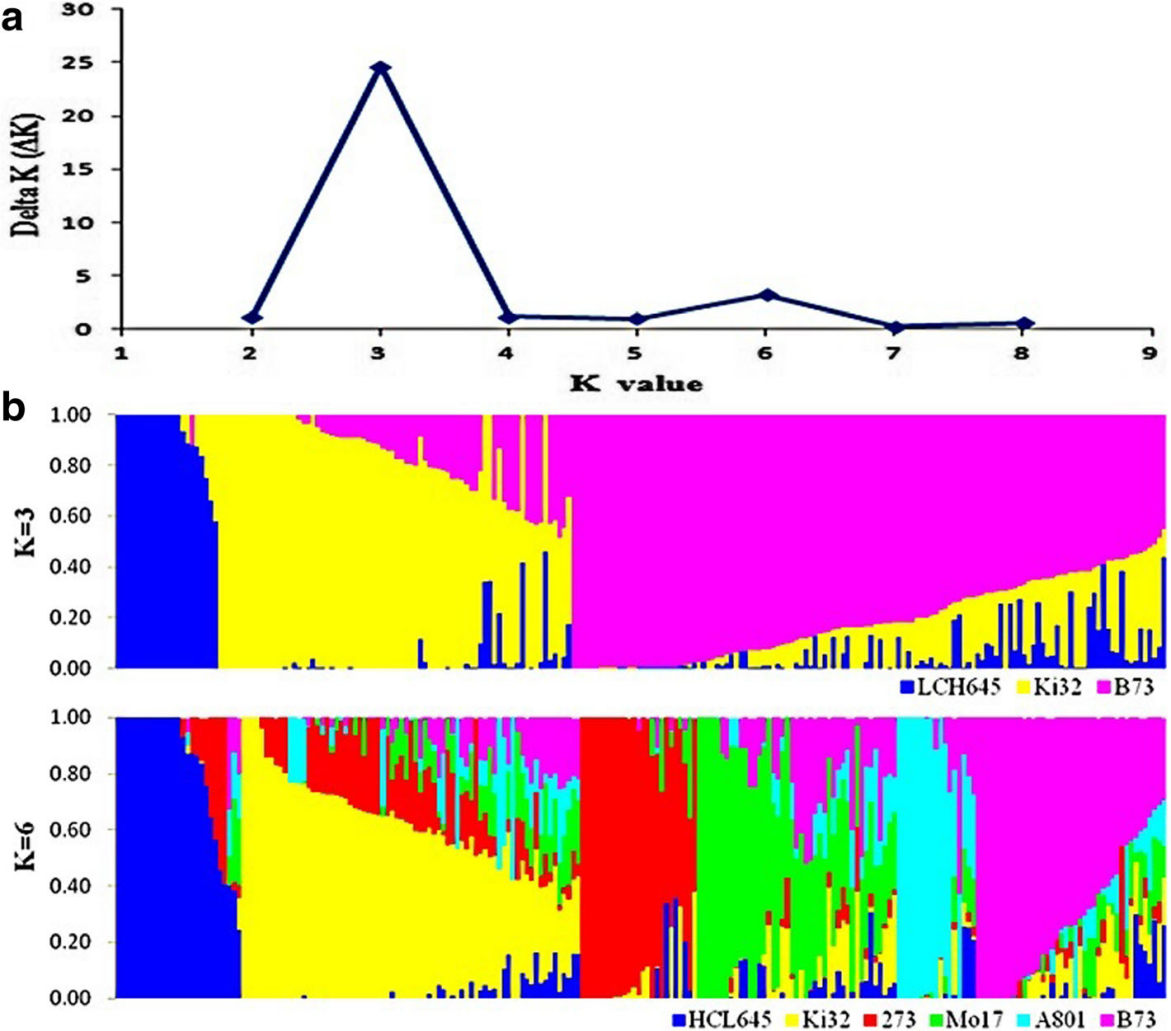
Model-based population structure division. Delta K was calculated from k = 2 to k = 8 (**a**). **b** showed the population structure division of the 226 accessions by using 8905 SNPs

The most significant peak in *ΔK* was observed (Fig. 1a) when k was three. This suggests that this panel of maize germplasm could be divided into three groups (Fig. 1b), with representative lines of HCL645, Ki32, and B73, respectively. These results were consistent with the experimental breeding according to the pedigrees and knowledge of breeding history. Twenty-seven lines were included in the HCL645 group, with most lines derived from HCL645. Seventy-eight lines were contained in the Ki32 group, with many lines derived from Ki32, or accessions introduced from tropical regions. The B73 group contained 120 lines, mainly being derived from B73 and its descendants. Furthermore, when k is six, the second peak of *ΔK* was observed, meaning that this panel could be further divided into six subgroups, with representative lines of HCL645, Ki32, QR273, Mo17, A801, and B73, respectively (Fig. 1a). The other lines with membership probabilities < 0.5 were appointed into one cluster called the mix group.

**Fig. 2:**
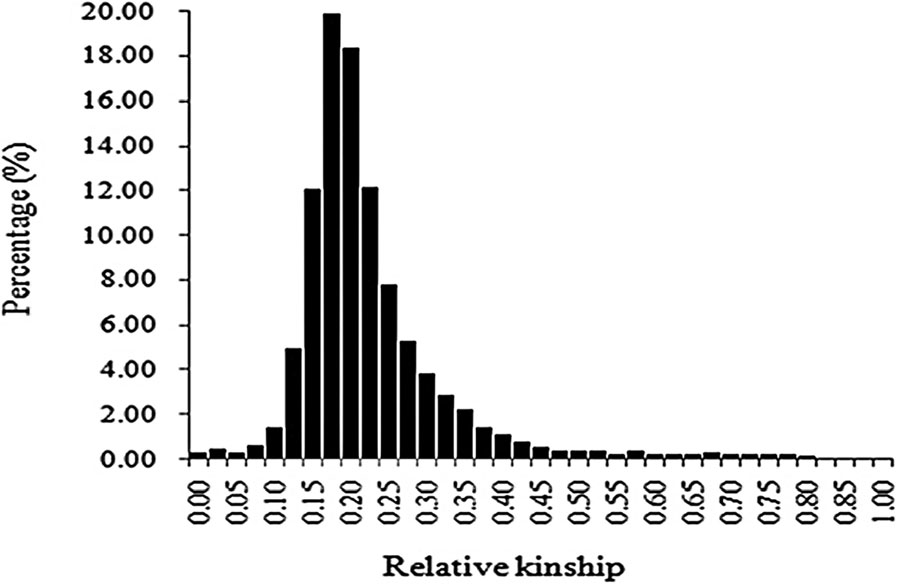
The distribution of paired relative kinshipamong226accessions. Values were calculated through TASSEL software 5.2.31 package by using 43,980 SNPs

Estimation of relative kinships showed that, in this panel: 82.8% of paired relative kinship ranged from 0.05 to 0.28, 0.33% of paired relative kinship equaled to 0, 0.38% of paired relative kinship ranged from 0 to 0.05, and the remaining ranged from 0.30 to 0.80 (Fig. 2). This analysis indicated that a weak or various relative kinship existed in this collection of elite maize germplasm, which was consistent with known pedigree/sources of the 226 lines.

### Neighbor-jointing tree and principal component analysis

**Fig. 3:**
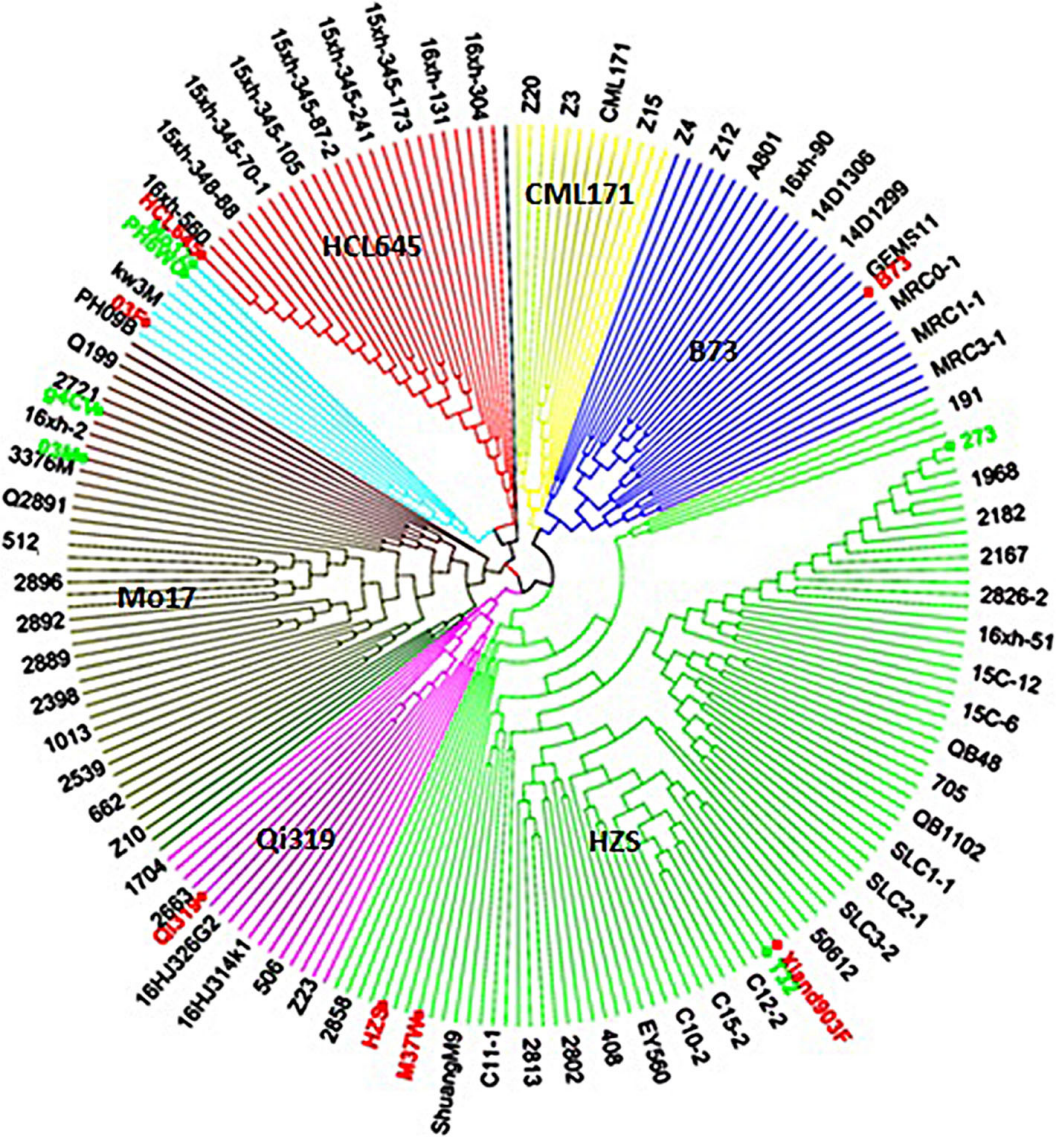
Neighbor-joining trees of the 226 maize accessions. Mo17 is a representative inbred line of Non-Stiff Stalk (NSS). Qi319 is a representative inbred of the Ludahonggu group. ‘HZS’ is an abbreviation of Huangzaosi, which is a representative inbred of the Tangsipingtou group (TSPT). B73 is a representative inbred of Stiff Stalk Synthetic (SS). CML171 is one representative inbred of tropical/subtropical group. In addition, HCL645 is one representative inbred of Monsanto

**Fig. 4:**
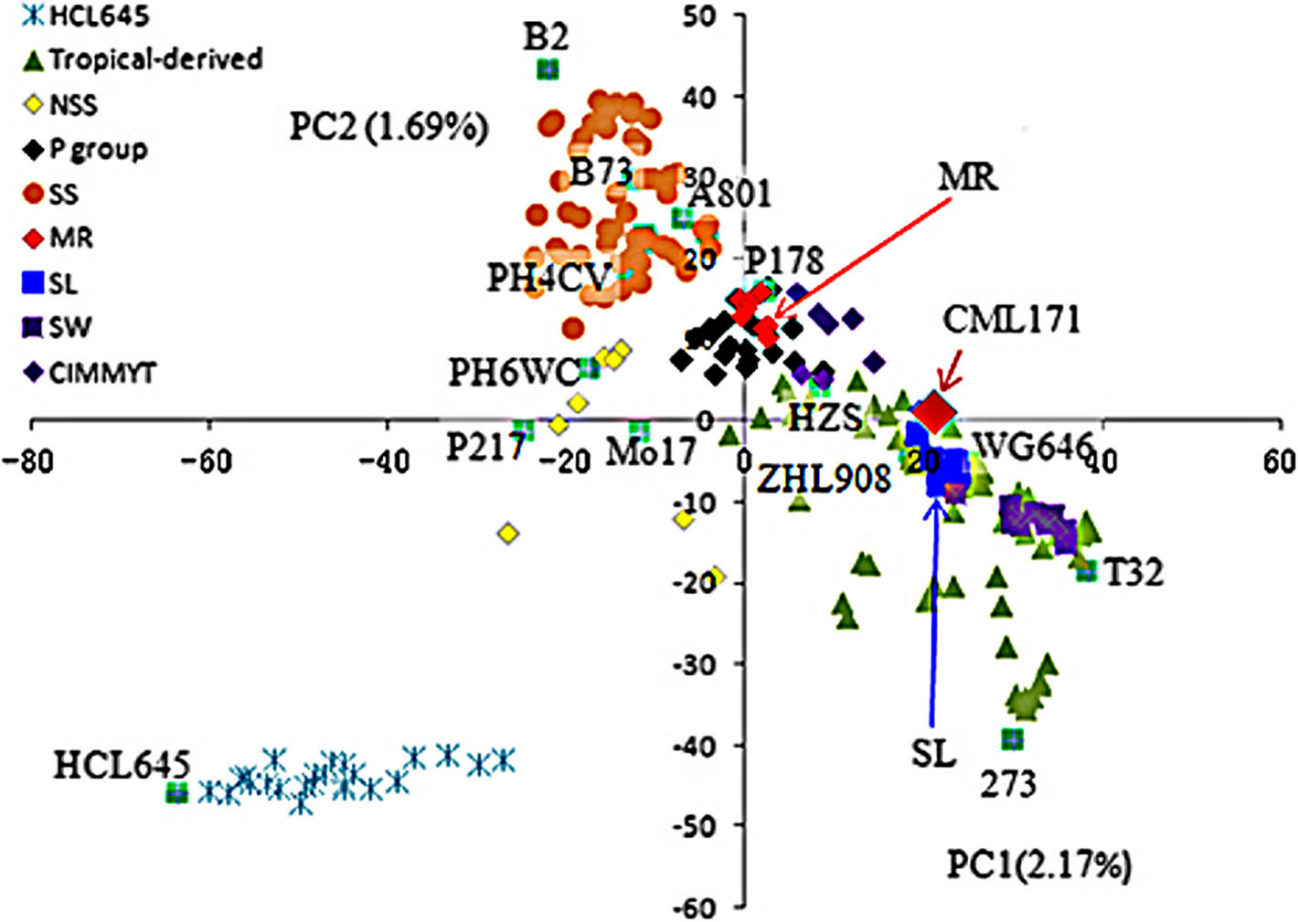
Results of principal components (PCs). ‘HCL645’ is one representative line of Monsanto accessions. ‘Tropical-derived’ means accessions containing tropical genes in their pedigrees. ‘NSS’ means Non-Stiff Stalk (NSS) subgroup. ‘SS’ means Iowa Stiff Stalk Synthetic group. ‘MR’ means accessions derived from the generations of Tuxpeño crossing with SS germplasm. ‘SL’ means accessions derived from the generations of Suwan crossing with Lancaster germplasm. ‘CIMMYT’ means accessions come from Maize and Wheat Improvement Center (CIMMYT)

A neighbor-joining tree was constructed based on the modified Euclidean distance and is shown in Fig. 3. The 226 accessions were clustered into six major groups according to their origins, with representative lines of CLH645, CML171, B73, HZS, Qi319, and Mo17 (Fig. 3). The Huangzaosi (HZS) cluster contained 87 inbred lines, with lots derived or included Suwan genetic material in their pedigrees. The B73 cluster contained 29 inbred lines, the Mo17 cluster contained 44 lines, the HCL645 cluster contained 27 lines, the CML171 cluster contained 13 lines, and the Qi319 cluster contained 17 lines. These results showed moderately common ground with some difference compared to that evaluated by using model-based method. The principal component analysis (PCA) results showed obvious population differences among HCL645, Iowa Stiff Stalk Synthetic (SS), tropical-derived lines, P groups, and Non-Stiff Stalk (NSS) subgroups, with representative lines including HCL645, B73, Ki32, P178, and Mo17. Lines derived from hybrid offspring of the Suwan (SW) population crossing with NSS population were pointed into tropical-derived subgroup, with smaller distance to NSS lines when comparing with that between Suwan and NSS lines. Lines derived from hybrid offspring of the Tuxpeño population crossing with the Reid population were distributed in the middle of tropical-derived population and the SS population (Fig. 4).

### Linkage disequilibrium (LD) level and genetic diversity

**Fig. 5:**
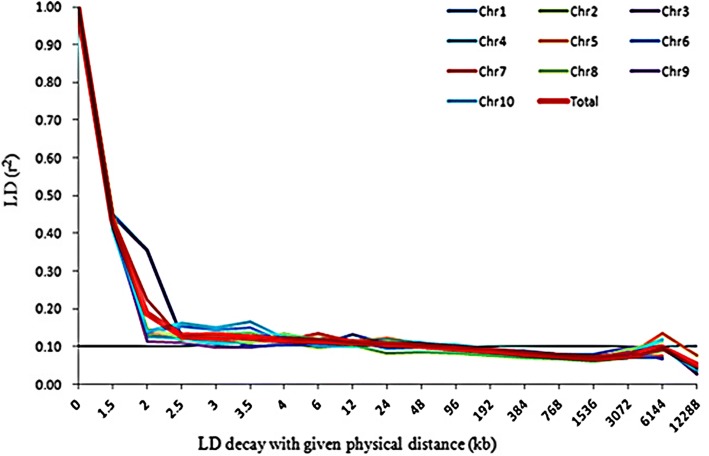
Linkage disequilibrium (LD) level. ‘Chr1-Chr10’ means chromosome 1 to 10. ‘Total’ means the whole genome

Linkage disequilibrium evaluation results showed that the average LD level is 41.15 kb in the panel of 226 lines and varies from 3.5 to 96 kb across 10 chromosomes (Chr.), with 3.5 kb on Chr.1, and 96 kb on Chr.5, Chr.6 and Chr.10 (Table 1; Fig. 5). For the whole panel of 226 lines, a total of 293,060 alleles were detected. The average gene diversity was 0.67, varying from 0.50 to 0.84, and the average PIC was 0.60, varying from 0.38 to 0.82.

**Table 1:** Average LD level of the 10 chromosomes for r2 greater than 0.1

Chromosome	Linkage disequilibrium (LD) (Kb)
1	24
2	6
3	3.5
4	6
5	96
6	96
7	48
8	12
9	24
10	96
Average	41.15

## Discussion

### Specific subpopulation structure division was found in this panel

Population structure analysis is usually a primary task in maize breeding research. When using multiple panels with different genetic backgrounds, some inconsistent results were reported. In China, four main heterotic groups are always clarified, with representative inbred lines of Huangzaosi, Ye478, Mo17, Lv28, and P178 [24, 26]. In the United States, NSS and SS are two mainly heterotic groups that play important roles in maize breeding both historically and currently (van [22]). When joining tropical and temperate maize accessions into one panel, two subgroups are always divided first, and then much more complex population structure differentiation information is revealed in-depth [11, 26, 27]. In this study, three subgroups are divided first, with representative lines selected as HCL645, Ki32, and B73, respectively (Fig. 1). Most lines belonging to the subgroup Ki32 were selected directly from the landrace of the Suwan population, or contained Suwan blood in their pedigrees. For instance, an inbred line of Ki32 is selected from the landrace of Suwan1 population, directly. QB446 was selected from the crossers of the Suwan1 population and Mo17. The two lines were also appointed into the same subgroup of Ki32. The heterotic mode of crossing lines from the subgroup of B73 with lines from the subgroup of Ki32 is widely used for maize breeding in the southern Chinese environment. Some excellent hybrids were released and widely used for maize production in southern China, such as Jinyu838 with parental lines QB446 and QB1018, wherein QB1018 belongs to the subgroup of B73 and QB446 to the subgroup of Ki32. In addition, one novel subgroup of HCL645 was identified, which contained many lines with HCL645 in their pedigrees. Dika517 is one important hybrid with HCL645 in one parental line: it was one example of a heterotic mode crossing lines belonging to the subgroup of HCL645 with lines belonging to the subgroup of B73. Dika517 was widely cultivated in the northern parts of China. However, lines from the subgroup HCL645 also showed higher combined abilities when crossing with lines from the subgroup of Ki32. For instance, crosses derived from 15xh-345-59 and 50,612 are compared in regional maize testing for the second time, and showed better performance in tropical or subtropical environments. 15xh-345-59 belongs to the subgroup of HCL645, and 50,612 belong to the subgroup of Ki32. These results showed abundant proof of maize population structure differentiation, which provides new insight for maize germplasm improvement in tropical or subtropical regions.

### Abundant genetic diversity existed in this panel

Knowledge of genetic diversity among accessions is indispensable in maize research and breeding programs, which provide scientific proof for the evaluation of detection resolution for specific panels and quantitative traits [8]. For one panel, significantly more genetic diversity would show stronger population structure differentiation, lower LD levels, and weaker pair-relationships. Research showed that different population compositions would impact genetic diversity. Tropical maize germplasm always showed much more genetic diversity, lower LD levels, and weaker pair-relationships [28]. In this study, lower LD levels were found, with average LD decay distance to be 41.15 kb, varying from 3.5 to 96 kb (Table 1 and Fig. 5). This was much smaller than that when using elite temperate maize germplasm only, with average LD decay distance at 328 kb, varying from 272 to 595 kb [26]. However, the LD levels found in this study were also larger than that identified in Yan’s report, with average LD levels at 5–10 kb, varying from 1 to 10 kb [28]. One possible reason is that in Yan’s panel, many original tropical accessions were included, and much more genetic diversity existed. However, in this study, where many improved tropical lines were derived from tropical populations and some local landraces, the tropical blood ratio was lower. In addition, gene diversity (GD) of 0.67 and PIC of 0.60 was identified in this study, which was obviously higher than that reported before, with GD and PIC ay 0.36 and 0.29 [26]. One possible reason for this phenomenon is that much more tropical maize germplasm were included in this study. In our previous research, 367 elite lines were collected. Because of the narrower genetic base among lines, stronger pair-relationships existed, with 94.97% of paired relative kinship ranging from 0.05 to 0.28 [26], which was significantly higher than that detected in this study, with 82.8% of paired relative kinship varying from 0.1 to 0.4 (Fig. 2). Because of this, we integrated maize germplasm from Suwan and temperate regions together in this study, wherein the Suwan population showed much more abundant genetic diversity than that of temperate populations [12, 27]. These results showed that the panel used in this study contained more abundant genetic diversity, along with lower LD levels, which would provide higher detection resolution for using genome-wide association study to dissect the variation in complex quantitative traits in maize.

## Conclusions

In this study, we clarified the population structure differentiation of maize germplasm derived from Suwan germplasm and related improved lines. Two novel subgroups were found, with representative lines identified as Ki32 and HCL645, respectively. However, much more genetic diversity and lower LD level were also detected, which revealed that this panel could be used for the dissection of quantitative traits’ genetic variation by using the genome-wide association study (GWAS) strategy. These results would provide some additional information for heterotic group division and use. As a result, this may facilitate better maize breeding, especially in tropical and subtropical regions.

## Materials and methods

### Plant material

The maize panel used in this study contained 226 accessions derived from the Suwan population and temperate resources. These contained 98 temperate accessions, with representative lines of B73 and Mo17, which were widely used in maize breeding historically [10]. In addition, most temperate lines were used in the latest maize breeding program in China, such as QB1013 and HC3. In addition, 128 accessions derived from the Suwan population were also selected, such as Ki32, QR273, and QB446, all of which are widely used in maize breeding practices in the southwest of China. Detailed information is listed in Additional file 1: Table S1.

### Genotyping and quality evaluation

This panel was genotyped by using MaizeSNP50 BeadChip, including 56,110 SNPs. Those SNPs were evenly distributed across the maize genome based on the B73 reference sequence (www.illumina.com/maizeSNP50). Leaves were collected from the five-leaf stage of maize seedlings. The tip of young and tender maize leaves of five plants were collected as a bulk to extract genomic DNA, according to the modified CTAB procedure [18]. Then, 200 microgram (μg) genomic DNA of each sample was selected to do further analysis. DNA quality assessment and genotyping work was done at the Beijing Compass Biotechnology Company, according to the Infinium® HD assay ultra-protocol guide. A total of 54,976 of 56,110 SNPs were called successfully among the 226 lines. SNPs with a missing rate of more than 20% and minor allele numbers (MAF) of < 0.05 were excluded from the genotyping dataset. Then, the source sequences of remaining SNPs were identified through BlastN searches against the reference genome sequence of B73 (RefGen-V1, http://www.maizegdb.org/). SNPs with ambiguous physical positions or multiple blast-hits were excluded from the genotyped dataset. After that, 43,980 high-quality SNPs were used in further analysis.

### Model-based population structure analysis

In total, 8905 SNPs with high genetic diversity, low missing rates, and even distribution across the genome were selected to investigate the population structure differentiation of the 226 lines, by using the model-based approach [24]. Structure V2.3.3 software [6] was run with k (the number of populations) from 1 to 15, with five runs for each k, with a burn-in period of 10,000 and 10,000 replications. The ad hoc statistic delta K (∆K) was used to determine the number of clusters [3]. Outputs of Structure were integrated by using CLUMPP software [7]. Lines with membership probabilities of more than 0.5 were assigned to corresponding clusters when incorporation with pedigree information for each lines [26].

Construction of neighbor-joining tree and principal component analysis (PCA).

To obtain an in-depth picture of the genetic relationships among 226 inbred lines, the genetic distance between pair-lines was calculated by using 43,980 SNPs. Modified Euclidean was defined as follows: D = 1 - identity by state (IBS) similarity [8]. IBS means the probability that alleles derived at random from two individuals at identical loci are the same. For any two lines, the probability of IBS was averaged over all non-missing loci. Then, the cladogram was constructed based on the distance matrix described above, by using the neighbor-joining (NJ) algorithm [30]. Clusters were observed from the result of the phylogenetic tree. All calculations were carried out in TASSEL software 5.2.31 [2]. PCA of 226 lines was done according to the Patterson’s description [15] using TASSEL software 5.2.31 [2].

### Linkage disequilibrium (LD) evaluation

By using 43,980 SNPs with MAF > 0.05 and missing data < 20%, pair-SNPs’ LD level was evaluated using the squared Pearson correlation coefficient (r^2^) between vectors of SNP alleles according to the method described by Hill [5]. The cutoff value of r^2^ was set to be 0.1 [28]. TASSEL 5.2.31 software was run with a 20 kb slide-window size [7], the spacing between two loci on the same chromosome was segmented in distance bins of 50 kb, and the average LD was assessed for each bin [16].

### Summary statistic of 226 lines

The pair relationship among the 226 inbred lines was estimated by definition as follows: F_ij_ = (Q_ij_ – Q_m_/(1 – Q_m_), where Q_ij_ is the probability of IBS for random loci from i and j, and Q_m_ is the average probability of IBS for loci from random individuals. Pair-kinship coefficients of 0, 0 to 0.1, and 0.1 to 0.5 indicated weak, intermediate, and strong similarity between accessions, respectively [29]. The gene diversity (GD) and polymorphism information content (PIC) were calculated using PowerMarker V3.25 [9], with GD as the probability that two alleles randomly chosen from the test sample were different [11]. The PIC was calculated as follows: ${\widehat{\mathrm{P}\mathrm{IC}}}_{\mathrm{l}}=1-\sum \limits_{\mathrm{u}=1}^{\mathrm{k}}{\overset{\sim }{\mathrm{P}}}_{\mathrm{l}\mathrm{u}}^2-\sum \limits_{\mathrm{u}=1}^{\mathrm{k}-1}\sum \limits_{\mathrm{v}=\mathrm{u}+1}^{\mathrm{k}}2{\overset{\sim }{\mathrm{P}}}_{\mathrm{l}\mathrm{u}}^2{\overset{\sim }{\mathrm{P}}}_{\mathrm{l}\mathrm{v}}^2$, where *plu* and *plv* are the frequencies of the *u*th and vth alleles for marker l, respectively.

## Additional file

Additional file 1: **Table S1.** Summary information of the 226 accessions. (XLS 25 kb)

## Abbreviations

∆K: delta K
BSSS: Stiff stalk
Chr: Chromosomes
CIMMYT: Maize and Wheat Improvement Center
GBS: Genotyping by sequencing
GD: Gene diversities
GWAS: Genome-wide association study
HZS: Huangzaosi
IBS: Identity by state
LD: Linkage disequilibrium
MAF: Minor allele numbers
NSS: Non-Stiff Stalk
PCA: Principal component analysis
PIC: Polymorphism information contents
SNP: Single-nucleotide polymorphisms
SS: Iowa Stiff Stalk Synthetic
SW: Suwan
